# Generalization of Object Localization From Whiskers to Other Body Parts in Freely Moving Rats

**DOI:** 10.3389/fnint.2019.00064

**Published:** 2019-10-31

**Authors:** David Deutsch, Elad Schneidman, Ehud Ahissar

**Affiliations:** Department of Neurobiology, The Weizmann Institute of Science, Rehovot, Israel

**Keywords:** whisker, localization, tactile, haptic, active touch, sensory history, decision

## Abstract

Rats can be trained to associate relative spatial locations of objects with the spatial location of rewards. Here we ask whether rats can localize static silent objects with other body parts in the dark, and if so with what resolution. We addressed these questions in trained rats, whose interactions with the objects were tracked at high-resolution before and after whisker trimming. We found that rats can use other body parts, such as trunk and ears, to localize objects. Localization resolution with non-whisking body parts (henceforth, ‘body’) was poorer than that obtained with whiskers, even when left with a single whisker at each side. Part of the superiority of whiskers was obtained via the use of multiple contacts. Transfer from whisker to body localization occurred within one session, provided that body contacts with the objects occurred before whisker trimming, or in the next session otherwise. This transfer occurred whether temporal cues were used for discrimination or when discrimination was based on spatial cues alone. Rats’ decision in each trial was based on the sensory cues acquired in that trial and on decisions and reward locations in previous trials. When sensory cues were acquired by body contacts, rat decisions relied more on the reward location in previous trials. Overall, the results suggest that rats can generalize the idea of relative object location across different body parts, while preferring to rely on whiskers-based localization, which occurs earlier and conveys higher resolution.

## Introduction

Object localization is a basic task in all perceiving agents, living or artificial. In living agents, this is an active and dynamic task – animals move their sensory organs while localizing objects around them (Uexkull, 1926; Kleinfeld et al., 2006; Diamond et al., 2008). When rats explore their environment, they use a combination of body, head and whisker movements to effectively comb the environment for interesting features. When encountering an object, its location may be coded in several egocentric frames of references (Ahissar and Knutsen, 2008; Knutsen and Ahissar, 2009). When the rat chooses to explore that object with different sensory modalities (e.g., moving from macrovibrissa-based sensing to nostril-mouth-microvibrissa sensing), a transfer between these frames of references is required. Rats typically implement such transfers immediately – within one sensory sampling cycle – suggesting that these frames of references are continuously linked (Sherman et al., 2017).

Whisker-based object localization was extensively studied during the last two decades, using a variety of experimental approaches and addressing various coding and processing aspects. Most of the studies had been limited to object location within the horizontal plane of a row of whiskers, a plane that can be spanned by two variables. The most natural set of variables are the whisker-centric polar coordinates: azimuth and radial distance (Knutsen and Ahissar, 2009; Ahissar and Knutsen, 2011). It turned out that the vibrissal system can code these variables in more than a single way (Cheung et al., 2019). Two plausible coding schemes are the so-called orthogonal and morphological schemes. In the orthogonal scheme, a single neuron can in principle code the two coordinates via its firing rate (coding radial distance) and firing phase (within the whisking cycle, coding azimuth) (Szwed et al., 2003; Szwed et al., 2006; Curtis and Kleinfeld, 2009; Knutsen and Ahissar, 2009; Ahissar and Knutsen, 2011; Kleinfeld and Deschênes, 2011). In the morphological scheme, the object’s coordinates in the horizontal plane are represented by the coordinates of a morphological plane of two whisker variables, one motor and one sensory (Ahissar and Knutsen, 2011; Bagdasarian et al., 2013). Several variables can be used, where those demonstrated experimentally are related to the whisker angle (motor) and whisker curvature (sensory) (Solomon and Hartmann, 2006; Birdwell et al., 2007; Towal et al., 2011; Boubenec et al., 2012; Bagdasarian et al., 2013; Pammer et al., 2013; Quist et al., 2014; Campagner et al., 2016, 2018, 2019; Severson et al., 2017). Both of these coding schemes can be actively controlled by the vibrissal system via the modulation of the relevant motor variables (Ahissar and Knutsen, 2011; Bale et al., 2015; Voigts et al., 2015; Ahissar and Assa, 2016; Sherman et al., 2017).

The mechanisms with which rodents localize objects in their daily life are not known. In the laboratory, rats and mice are often trained to localize objects and report their location (reviewed in Ahissar and Knutsen, 2008; Knutsen and Ahissar, 2009; O’Connor et al., 2010; Kleinfeld and Deschênes, 2011; Bush et al., 2016). Two major constrains limit the interpretation of the results of laboratory localization experiments. First, in most experiments rodents’ perceptual behavior has been constrained in some way, ranging from rigid head fixation (Deutsch et al., 2012; Pammer et al., 2013; Moore et al., 2015; Urbain et al., 2015; Cheung et al., 2019) to requiring nose-poking at a fixed position (Knutsen et al., 2006). Second, training may guide the animals to use strategies that are specific to the trained environment and protocol. Here we ask how do animals perform a localization task when both whiskers and other body parts are available, using two different training paradigms.

We developed an apparatus that allows rats to freely move their body, head and whiskers during localization. We employed two different training procedures, one optimized for revealing localization threshold (using a staircase protocol) and another optimized for comparing localization success at different location offsets (using a block design). The task was a relative bilateral localization task (Saraf-Sinik et al., 2015). Training the animals using two different learning paradigms, and two different whisker trimming sequences allowed us to assess the dependency of task generalization on perceptual experience.

Tracking head and whisker motion at high-speed (500 Hz) allowed the assessment of the coding scheme used by the rat in each condition. Given the task’s coordinates and the body parts we examined, we focused here on two major coding aspects: temporal (relevant for the orthogonal vibrissal scheme) and spatial (relevant for the morphological vibrissal scheme) – aspects that could be assessed for all measured body parts. Trimming whiskers and manipulating the spatial arrangements of the poles allowed the determination of the use of body parts other than whiskers for localization, as well as the dependency of localization on the absolute location of the poles. The results presented here suggest that rats are flexible in selecting localization strategies, although they exhibit clear preferences.

## Materials and Methods

### Animals

Fourteen female albino rats (Wistar) were trained in a two-alternative forced-choice task to discriminate the relative horizontal offset of two vertical poles. Training commenced at the age of 7 weeks. Rats were held 2 in a cage, under a 12 h light-dark cycle. Experiments were done during the light cycle. Water was withheld for 20–24 h before each training session. Fruit juice (mango-flavored) was given as reward during training, and unlimited water was available throughout the following 20–24 h. Solid food was available *ad libitum*, except during training. Four groups of rats (groups 1–4, see Table 1) were trained, and the training protocol differed between groups in three ways: the object geometry (Wide/Narrow), the training paradigm (Block/Staircase), and the trimming procedure (see Table 1 and more details below under ‘Behavioral training’). All rats were first trained with a full pad of whiskers (‘full’ in Table 1). In groups 1, 2, 4, whiskers were trimmed from a full-pad to a single row of whiskers (‘row C’ in Table 1), to whisker C2 only (‘whisker C2’ in Table 1) to no-whiskers. In group 3, whiskers were trimmed directly from a full pad to no-whiskers. Note that single whisker/row means single whisker/row in each side of the face, throughout the paper. Trimming (from full to row C, from row C to whisker C2, from C2 to no-whisker or directly from full to no-whisker) was done when the animal showed stable performance in the previous condition. That is, a stable percent of correct trials per offset in the block design, and a stable threshold offset in the staircase paradigm. Trimming was repeated throughout data collection period every 2 days, and at least 1 day before the experiment. All experimental protocols were approved by the Institutional Animal Care and Use Committee of the Weizmann Institute of Science.

**TABLE 1:** Summary of animal groups used in this study.

**Group**	**Geometry**	**Training protocol**	**Trimming protocol**	**Rats**	**Sessions**	**Trials**	**Figures/movies**
1	Narrow	Staircase	Full → row C → whisker C2 → No whisker	2	32/14/20/18	1468/637/1131/1156	1A,C (top), 2A–C, 3E, 4C–F, 5A
2	Narrow	Block	Full → row C → whisker C2 → No whisker	6^∗^	9/14/96/19	18/91/6714/951	1C (bottom), 3A,C,D, A,B, 5A–I, 6A,B, S1A,B
3	Wide	Staircase	Full pad → No whiskers	3	110/4	7472/243	3B
4	Wide	Staircase	Full → row C → whisker C2 → No whisker	3	150/241/169/71	9469/14615/9617/3974	1B, 6C,D, S1A,B

*^∗^For three out of the six rats in group 2, once approached a stable performance with a single whisker, only 17mm offsets were used, but at three positions in the posterior/anterior axis (Figure 5F, see section Materials and Methods).*

### Experimental Apparatus

**FIGURE 1:**
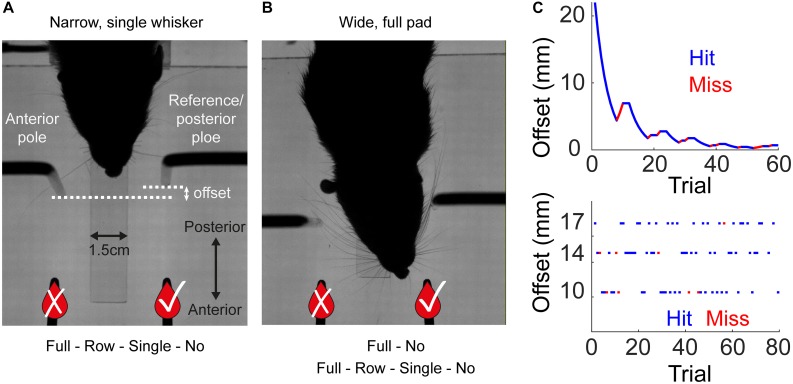
Experimental setup and training procedure. Rats were trained to associate the spatial arrangement of two vertical poles with a reward side. **(A)** A rat from Group 1 (Narrow, Staircase; see Table 1) with a single whisker during task performance, top view. The reward (the sipper marked with V) was always given at the reference (posterior) pole side. In the Narrow configuration (Groups 1–2, see Table 1), the pole-sipper geometry was designed such that rats contacted the poles with their body on the way to the sippers. For all rats that were trained in the Narrow configuration, whiskers were trimmed gradually from a full pad to a single row (row C) to a single whisker (C2) to no whisker (Full-Row-Single-No). **(B)** The Wide configuration (Groups 3–4, see Table 1) was designed such that rats did not need to contact the poles with their body on the way to the sippers. In this configuration the distance between the poles is larger in the left-right axis, and the poles are closer to the sippers in the posterior anterior axis [compare **(A)** and **(B)**; see section Materials and Methods for more details]. In Group 3, whiskers were trimmed from a full-pad to no-whisker (Full-No), while in Group 4 whiskers were trimmed gradually (Full-Row-Single-No). **(C)** Each rat was trained in one of two paradigms. In the Staircase paradigm (top; Groups 1, 3, 4) the offset in a given trial was chosen based on animal performance in previous trials. In the Block paradigm (bottom; Group 2) the offset in each trial was chosen randomly from three possible offsets in a given session, regardless of rat’s performance. The side of the reference pole (left or right) was chosen randomly (Block training) or pseudo-randomly (Staircase training) between trials (see section Materials and Methods). An example session is shown for each training paradigm. Hit trials are marked in blue, miss in red.

The setup used in this study is identical to the one described in Saraf-Sinik et al. (2015). Briefly, in an acoustically isolated and lightproof chamber, the setup included two distinct areas: “arena” and “task area,” connected by a motorized door. The general sequence of a single trials (see example trial in Supplementary Movie S1) was as follows: upon door opening, the rat entered the task area to perform a trial. The rat walked on a plastic bridge (1.5 × 12 cm), between two vertical poles (2.8 mm diameter), and made a left or right choice. Following a correct choice (licking of the correct metal drinking tube – ‘sipper,’ see Figures 1A,B), rat was rewarded with mango juice. Licking was detected by a capacitance-based detector, triggering reward delivery. Next, the rat was called back into the arena via signaling of a second reward (‘side reward’) and upon returning to the arena, the front door closed. Motorized doors controlled the passage between spaces and IR beam sensors reported crossing through. Each trial, the motors moved to a pre-defined resting position before moving to the next position to minimize auditory cues. Rats performed ∼60 trials per session (∼40 min; see Table 1), consuming ∼10 ml juice. A session was terminated, when rats stopped performing the task for three consecutive trials. High-speed camera (MotionPro; Redlake) located above the task area (‘top camera,’ example Supplementary Movies S2–S8) recorded videos of the rat performing the task at high spatiotemporal resolution (640 × 880, 500 Hz). Another infrared-sensitive CCD camera (55–701; Edmund Industrial Optics) allowed monitoring of behavior in the arena (Supplementary Movie S1). IR backlight led array (830 nm, MB-OBL9 × 9-IRN-24-1 V OmniLight flat dome; Metaphase Technologies) illuminated the task area, and a 10 × 10 cm array of infrared (940 nm) LEDs (L940-04AU; Epitex) illuminated the arena. None provided visible illumination for the rats, which performed the task in complete darkness, but provided illumination visible by the cameras’ sensors. Each of the two poles (2.8 mm diameter), as well as the front sippers were mounted on *x-y*-axes computer-controlled mobile tables (FB075-150-1.0M4 Nanomotion Linear Stage; Abiry Technologies), which allowed *x-y* positioning of 1μm precision. Rats were trained in two possible geometrical configurations: Narrow (Groups 1-2, see Figure 1A and Supplementary Movies S2, S5–S8) or Wide (see Figure 1B and Supplementary Movies S3, S4). The Narrow geometry was designed such that rats always contact at least one pole with their body before reaching a sipper. In the Wide geometry, rats could reach the sippers without contacting any pole with their body (Figure 1B). In the Narrow configuration, the lateral distance between the poles was 42 mm and the reference pole to reward sipper distance was 42 mm or 64 ± 6 mm for groups 1, 2, respectively. In the Wide configuration, the lateral distance between the poles was 52 mm, and the pole-sipper distance was 34 mm. The distance between the entrance door and the reference pole was 55 and 80 mm for groups 1 and 2, respectively, except in trials with unfixed reference (see below), where the three door-to-reference distances where 38, 55, or 72 mm for the ‘proximal,’ ‘original’ and ‘distal’ positions, respectively. Before the first experiment in a given day, a calibration procedure was performed to ensure that a single drop of mango juice is equal to 0.6 ml in both sippers. This was done by applying 15 drops per sipper (using the same control software that was used during the experiment), measuring the amount of juice with a measuring container, and fixing the drop size in the software. Typically, 2-3 rounds of calibration per sipper were needed to converge to the exact amount.

### Behavioral Training

Rats were trained to associate between the relative location of two vertical poles (left pole/right pole) and the side of the rewarding sipper. The rewarding sipper was always at the side of the reference/posterior pole (Figures 1A,B, reward sipper marked with ✓). In each trial, the animal entered the task area, passed between the left and right poles (one of them being the ‘reference pole’), and licked from the left or the right sipper (rarely the rat made no choice – didn’t lick from any sipper before going back to the arena). Mango juice reward was given to the rat upon licking in the rewarding sipper. Then the rat reentered the arena to collect the side reward (given regardless of rat success and always at the same port). Failing to choose the rewarding sipper led to withdrawal of both front and side rewards and to an increased time delay of the following trial. Two different pole-sipper arrangements (Narrow/Wide) and two different training paradigms (Staircase/Block) were used for different groups as summarized in Table 1. Two groups of rats (Groups 1, 2) were trained in the Narrow pole/sipper arrangement, where the geometry forced the animals to body-contact at least one poles on the way to the sippers. Two groups where trained in the Wide arrangement, where rats could reach the sippers without contacting any pole with the body. Rats in Groups 1, 3, 4 were trained in a ‘Staircase’ protocol, while Group 2 was trained in a ‘Block’ protocol. In the Staircase protocol the horizontal offset was always at 22 mm in the first trial of the session and changed according to rat success. Following three correct choices in a row (or one correct choice in the first trial) the offset was multiples by 10^0.1^ (following Knutsen et al., 2006) for next trial (for example, changed from 22 mm to 17.475 mm). Following a single wrong choice, the offset was divided by 10^0.1^ (for example, changed from 13.88 to 17.475 mm), but could not exceed 22 mm. There was no limit on the smallest offset. The reward side (= which pole was more posterior) in the staircase paradigm was chosen semi-randomly to reduce animal bias, using the same rule as used in Knutsen et al. (2006), and described below.

Which pole was more posterior, was randomized only in the first 10 trials and chosen according to the following bias-correcting rules in subsequent trials. These rules took into account the responses that each rat made during the 10 preceding trials:

$$\begin{array}{c} \left|S-O\right|>\left|L-R\right|\Rightarrow \left\{\begin{array}{c} S>O\Rightarrow 0\\ S<O\Rightarrow S \end{array}\right\}\\ \left|S-O\right|<\left|L-R\right|\Rightarrow \left\{\begin{array}{c} L>R\Rightarrow R\\ L<R\Rightarrow L \end{array}\right\}\\ |S-O|=|L-R|\Rightarrow \;U\left(\left[L,R\right]\right)\; \end{array}$$

These rules compared the number of times a rat approached the same (*S*) or opposite (*O*) side as in an immediately preceding trial, in addition to the cumulative number of responses made to the left (*L*) or right (*R*) side. When the ratios of responses to same/opposite and left/right side were different, the relative offset of the poles (i.e., which was more posterior) was predetermined in the upcoming trial. When these ratios were equal, the poles were randomly arranged (with *U*([*L*,*R*] being a distribution of equal left and right probabilities). These bias-correction rules were chosen to offset the occurrence of prevalent stereotypical trends, such as persistent responses to the same side or right–left–right switching between subsequent trials. Bias correction is common practice with rats (Carvell and Simons, 1990; Shuler et al., 2002). We used four parameters computed across 10 consecutive trials, which made it difficult to predict the upcoming stimulus configuration. In the Block training protocol, the offset was chosen in each trial from three possible offsets, regardless of rat performance in previous trials. All possible offsets were 20/17/14/10/6 mm. In the first sessions 20/17/14 mm were used. Later, 17/14/10 mm were used, and in the last sessions in a given whisker configuration, after rats achieved stable performance, the offsets were 17/14/10 in the first half of the session, and 14/10/6 mm in the second half of the session. The reference pole side (= reward side) was chosen randomly in each trial. Three rats out of six in Group 2 (Narrow, Block), after reaching stable performance with a single whisker, were transitioned for the rest of the sessions to an unfixed-reference paradigm. Here, the offset was kept at 17mm, but the reference pole was positioned in one of three locations in the posterior-anterior axis: Proximal/original/distal (Figure 5F). This was done in order to test if the absolute pole-location (rather than only the relative offset) was used as a tactile cue. A total of 57,556 trials were performed in 967 sessions, giving an average of 59.5 trials per session (see Table 1 for more details).

### Data Acquisition and Analysis

Behavioral events (crossing through a door, licking of a sipper) were tracked via IR beam sensors and capacitance switches. Their respective time stamps were logged as was trial relevant information (e.g., arrangement’s ID, chosen sipper), for performance analysis. Video files of rats performing the task were acquired by the high-speed camera and automatically saved upon trigger using the MIDAS software. Head position was tracked offline using BWTT the biotact whisker tracking tool, an artefact of the eu framework 7 project biotact 215910^1^. The curving index (CI, Figures 5G,H) is defined as the ratio between the length of head trajectory, and the distance between the start (entrance) and end (sipper) points in the head trajectory. Curvature is zero at CI = 1 and increases with CI. In a subset of movies, whiskers were tracked offline using MATLAB-based software (WhiskerTracker; Knutsen et al., 2005), a semiautomatic head and whiskers tracker (Figure 5D). Manual video-based analysis produced onset and offset frames of whisker-pole and body-pole contacts.

### Statistics

**FIGURE 2:**
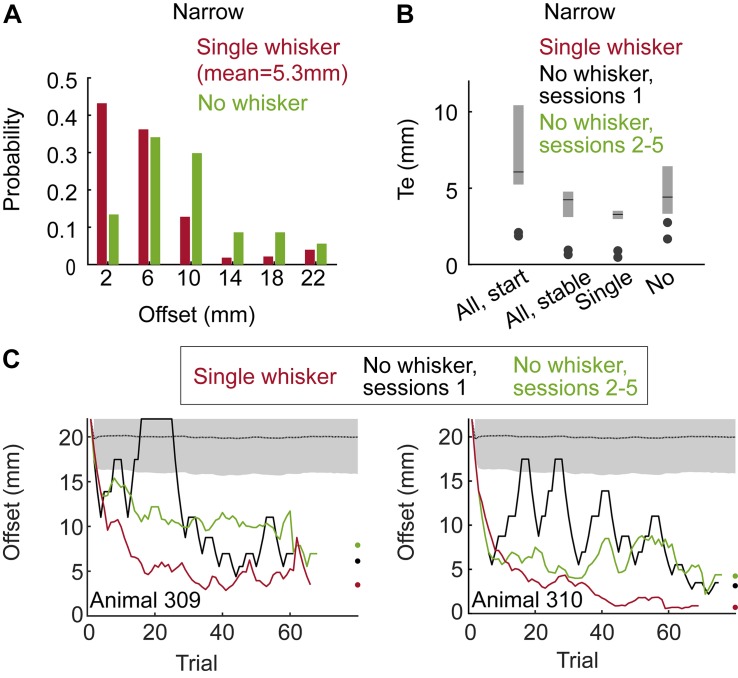
Localization by whiskers and body. **(A)** Distributions of localization thresholds in the single whisker (red) and no whisker (green) conditions (Group 1 - Narrow, Staircase). **(B)** Localization threshold (Te) for the two animals in Group 1. All-start/All-stable: sessions with all whiskers, start training (first sessions) and when behavior was stable (last sessions). Single/No – three sessions before/after trimming the last (bilateral) single whiskers. Te is defined as in Knutsen et al. (2006), see their Figure 10. Black lines indicate the median Te and the lower and upper end of the gray bars indicate the first and third quarantines, respectively. **(C)** Mean offset as a function of trial number for two animals. Means over the last 5 sessions with a single whisker (red), the first session after trimming (black) and the mean over sessions 2–5 after trimming (green). Black dotted line and gray shade are the mean and standard deviation for the offset of a rat performing at chance level (*N* = 5000 simulated sessions).

**FIGURE 3:**
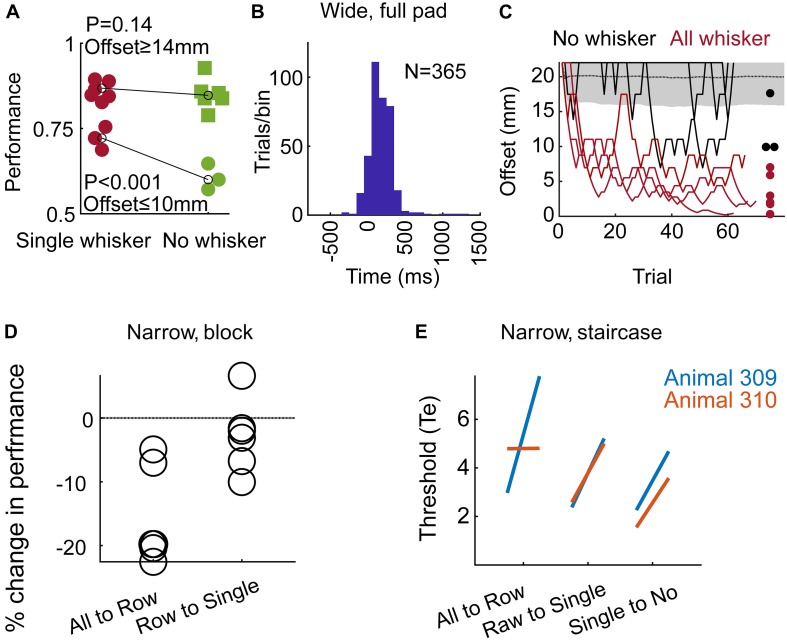
The use of whiskers and learning. **(A)** In the Narrow configuration, Block training paradigm (Group 2), performance is shown for large offsets (≥14 mm) and small offsets (≤10 mm). Black line – mean over all trials in the last 3 sessions before/after trimming (labeled in red/green as single/no whisker). Each symbol represents the rate of success of an individual rat. **(B)** Time from the Decision (turning) Point (see Results) to first contact between the body and any pole. Positive time means that the first body-pole contact occurred after the rat started the final turning. Group 3, full pad, Wide configuration, Staircase paradigm. **(C)** Two last sessions per rat before trimming (red), and first session after trimming (black) are shown for the three rats in Group 3. Dots represent the minimum offset in the session, after running a 10-trial moving average. Performance at chance level is shown as in Figure 2C. **(D)** Change in performance around trimming (comparing three sessions before/after trimming) for rats in Group 2 (Narrow, Block). Each symbol represents the percent of change in performance for an individual rat (comparing mean performance before and after trimming; negative value means drop in performance). Performance dropped for all six rats when trimmed from full-pad to a single row (‘All to Row’), and for 5/6 rats when trimming from a single row to a single whisker (‘Row to Single’). **(E)** Each line connects the mean localization thresholds over three sessions before and after each trimming step, for each rat.

**FIGURE 4:**
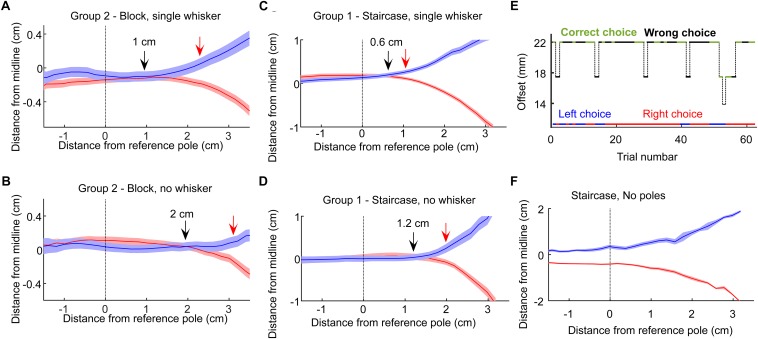
Decision points. **(A)** The mean and standard error of the head trajectories in left choice (blue) and right choice (red) trials for the rats in Group 2 (Narrow geometry, Block training). Data from the 3 sessions before trimming the single whisker. Solid arrow marks the last point where the left and right trajectories diverged. Red arrow marks the first point in which the difference between the two trajectories was larger than twice the sum of the standard errors from both trajectories. **(B)** Same as **(A)**, but for the 3 sessions after trimming the single whisker. **(C,D)** same as **(A,B)**, but at the Narrow/Staircase configuration. **(E)** A single session example from a control session. In control sessions the poles were removed from the setup. The pole base was still moving with the same logic (controlling for sound-based discrimination). Animal performance dropped to chance, with typically long epochs of repeated choice (here, a right-choice sequence is seen), showing that rats rely on tactile rather than auditory cue in the task. **(F)** Left/right head trajectories in the no-pole condition.

**FIGURE 5:**
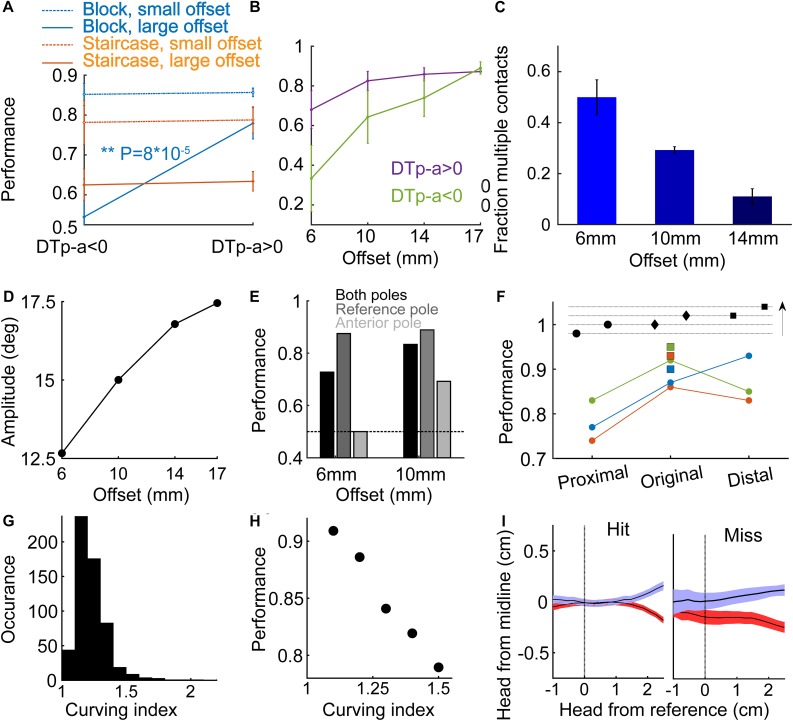
Temporal and spatial coding.**(A)** Mean performance for the Block-trained (Group 2, blue; Narrow geometry) and Staircase-trained (Group 1, red; Narrow geometry) rats, for small offsets (offset ≤ 10 mm for Block-trained, offset < median for Staircase-trained) and for large offsets (dotted line; offset ≥ 14 mm for Block-trained, offset > median for Staircase trained) rats. Performance is shown separately for trials with DTp-a < 0 (left) and for trials with DTp-a > 0 (right). **(B)** Mean performance for positive DTp-a (purple) and negative DTp-a (green), as a function of pole offset. **(C)** The fraction of trials with multiple (>1) whisker contacts with the reference pole. **(D)** Mean protraction amplitude in the whisker contacting the reference pole, as a function of offset. **(E)** Mean performance in trials where whiskers touched both poles (black), only the reference pole (dark gray) or only the anterior pole (light gray). **(F)** Further training in fixed offset. Top, illustration of poles arrangements: ‘original’ (middle, reference pole at the same location as during early training), ‘proximal’ (closer to entrance to the task area) and ‘distal.’ Arrow indicates the entrance-line to sipper-line direction. Each line represents the mean success rate of a rat during the first two sessions after moving to the changing-reference paradigm. Squares indicate the mean success rate of each rat in the last two sessions before moving to the non-fixed reference paradigm. **(G)** Curving index (CI, see section Materials and Methods) distribution. **(H)** Rat performance versus CI; mean over 6 rats and 581 trials. **(I)** Head trajectory (mean and standard error) for hit (left) and miss (right) trials.

**FIGURE 6:**
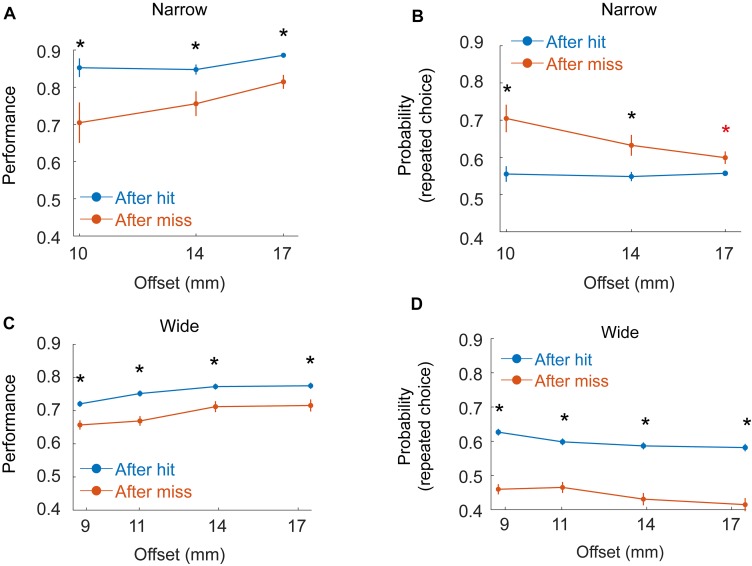
Dependence of decisions on previous trials. **(A)** Mean performance as a function of offset for six rats (Group 2 - Narrow, Block) given hit (blue) or miss (red) in the previous trial. Black asterisk indicate significant difference between hit/miss trials for a given offset after Bonferroni correction for multiple comparisons. **(B)** Probability for repeating the same choice as in the previous trial (left after left or right after right), given hit (blue) or miss (red) in the previous trial. Red asterisk indicates significance before but not after correction for multiple comparisons. **(C,D)** Same as **(A,B)**, but for the three rats in Group 4 (Wide, Staircase).

Estimated trajectory distributions (Figures 4A–D,F, 5I) are shown as arithmetic means ± SEM. Except when noted, comparisons between distributions were based on the parametric two-sample *t*-test, and a *p*-value of 0.01 or smaller was used as the cutoff for significance. The experimental threshold T_e_ in Figure 2B was calculated as in Knutsen et al. (2006), and in this case, to be consistent with the original report, Wilcoxon’s rank sum test for equal medians was used to compare the distributions. In Figure 3A the 4 offsets were divided into two groups: small offsets (6, 10 mm) and large offsets (14, 17 mm), and the statistical test was done between these two groups. In Figure 6, bootstrap was used for comparing the animal performance in after hit/miss trials for each offset. Bonferroni correction was used for multiple comparison. Black asterisk indicates significance after Bonferroni correction, while red asterisk (Figure 6B, 17 mm) indicates significance before but not after correction for multiple comparisons.

## Results

### Whisker and Body Were Both Used for Localization in Freely Moving Rats, but Whiskers Were Essential for Small Offsets

In a two-alternative forced choice task, rats were trained in a dark room to associate the reward side with the relative position of two vertical poles (Figure 1A). For a given group of rats, the reference pole was presented at a fixed location in the posterior-anterior axis (see Figures 1A,B), while the second pole, was presented in a more anterior location. The offset between the poles in the posterior-anterior axis was determined either randomly from a possible set of offsets (Block training) or based on animal performance in previous trials (Staircase training; see section Materials and Methods for more details). A reward was present in the reference pole side (reference side changed between trials randomly of pseudo-randomly, see section Materials and Methods), and given to the rat upon the detection of licking (see Supplementary Movie S1). Rats were trained in one of two training paradigms (Staircase or Block; Figure 1C and Table 1), and in one of two geometries (Wide or Narrow, Figures 1A,B and Table 1), see section “Materials and Methods” for more details.

We trained two rats using the Staircase protocol and Narrow configuration (Group 1, see Table 1 and Methods). In the Narrow configuration, rats were forced to contact both poles with their body before contacting a sipper in 100% of the trials, as a result of the horizontal distance between the poles in this configuration (see e.g., Supplementary Movie S7). Initially all whiskers were intact (‘full pad’). We then trimmed their whiskers in steps (full pad – single row – single whisker – no whiskers, see Table 1). Rats reached a mean offset of 5.3 mm across all trials in the last sessions with a single whisker (Figure 2A and Supplementary Movie S2). During trials with single rows or with single whiskers rats contacted the poles with whiskers in both sides in 99.2 or 99.5% of the trials, respectively. Trimming the last whisker increased the mean offset from 5.3 to 10 mm (*P* < 0.001, two-sample KS-test, Figure 2A). The drop in performance upon trimming the last whiskers indicates that whiskers were essential for the discrimination of offsets <6 mm. These whisker-dependent thresholds are similar to those obtained by rats required to nose-poke while discriminating (compare Figure 2B and Figure 10 in Knutsen et al., 2006).

Rats without whiskers (Group 1 – Narrow, Staircase) exhibited a rapid generalization process, transferring the localization task to non-whisker body parts, primarily the trunk and ears (Supplementary Movies S2, S4 show body-pole contact with/without prior whisker-pole contact, respectively; Supplementary Movie S8 shows an example trial with Narrow configuration and no-whiskers). Horizontal discrimination was evident already within the first session after trimming the last whiskers for both tested animals (Figure 2C). Their resolution, however, was on average poorer than that obtained with whiskers, increasing their median localization thresholds from 3.6 to 5.5 mm (Figure 2B; *p* = 0.02, Wilcoxon’s test). Interestingly, the performance was poorer in sessions 2–5 comparing to session 1 after trimming, suggesting that whisker-pole contacts are essential for maintaining the association between the pole arrangement and the reward side. This observation is consistent with the role of whiskers in learning, as shown below (see Figure 3). To further test the dependency of localization resolution on the use of whiskers or body we trained a group of six rats using the Block training paradigm (Group 2, Narrow configuration, see Table 1). In each session three offsets out of five possible offsets (20, 17, 14, 10, 6 mm) were used (first sessions with 20/17/14 mm, last sessions with 14/10/6 mm, see section Materials and Methods). In each trial, the offset was chosen randomly and independently of the (also randomly chosen) reward side. Same as in Group 1 (Narrow, Staircase), whiskers were trimmed in steps, from a full-pad, to a single row, to a single whisker to no-whisker (Full-row-single-no; see below the effect of trimming on performance in each step).

Trimming from a single whisker to no-whisker caused a significant decrease in performance for small offsets (Figure 3A; ≤10 mm, *p* < 0.001, two sample *t*-test), but not for large offsets (≥14 mm, *p* = 0.14, *t*-test). Interestingly, while whiskers were essential for offsets of 6 and 10 mm in Group 2 (Narrow, Block), they were essential for localization only for offsets of 6 mm or less in Group 1 (Narrow, Staircase), indicating experience-dependent body localization. In the Narrow configuration it was not possible to distinguish between intentional and accidental body contacts, nor to determine whether body contacts were used for the decision (see e.g., Supplementary Movie S7). What was clear is that the association between relative body contacts and the reward side was evident (Figures 2A–C, no-whisker condition; Supplementary Movie S8, no-whisker example). To test whether rats can generalize even with no previous experience of body-pole contact, we trained a third group of rats (Group 3 – Wide, Staircase, three rats; see Table 1) in a different pole-sipper geometry. Having a larger inter-pole-distance, and a shorter poles-to-sippers distance, rats could lick from a sipper without contacting a pole with the body (Figure 1B and Supplementary Movie S3). Indeed, rats with intact whiskers turned toward the left or right sipper before making any body-pole contact in 89% of the trials (Figure 3B; positive time means that the first body-pole contact occurred after the turning point). In this case, in the first session after trimming all whiskers at once (full-pad to no-whiskers), rat performance was not distinguishable from chance (Figure 3C, mean offset = 19.5 mm, *p* = 0.13). This result indicates that immediate transfer (i.e., within a session) from whisker- to body-based localization requires the association of body contacts with reward side before trimming the last whisker. Yet, we found that rats can learn to localize with body parts even without such pre-associations, as in the second day after trimming the mean offset (=10.5 mm) was significantly lower than that expected from chance behavior (*P* < 0.001). Discrimination threshold went up around each trimming step (except no change in rat 310, full-pad to single raw) for Groups 1/2 (Narrow, Block/Staircase, Figures 3D,E), and performance dropped for 6 or 5 rats when trimming from a full-pad to a single row or from a row to a single whisker, respectively (Figure 3D). This indicates that whiskers were used in the task during learning. Note that after training in a new whisker configuration, rats recovered to the previous threshold (Figure 3E).

### Rats Made Tactile Decisions Based on Whisker Contact When Whiskers Were Available

The finding that our rats could localize the poles without whiskers, already in the first session after trimming the last whisker (Figure 2), suggests that they associated body contacts with reward side already before the final trimming. In the Narrow configuration (Groups 1, 2), rats were forced to contact the poles with their body when approaching the sippers, allowing such association to merge during training. When having a single whisker, rats therefore faced the option of making a choice (left or right) based on whisker contact only or deciding only after contacting the poles with the body. To discriminate between these two scenarios, we looked at head trajectories in the three sessions before and after trimming. We found that in both training paradigms (Staircase and Block, Groups 1 and 2), rats made a decision 0.6–1 cm earlier when whiskers were available (Figures 4A–D).

Visual cues were not available, as the task was performed in dark (see section Materials and Methods). Olfactory cues were not available as juice was delivered only when the rat was licking the sipper (using a capacitance-based detector). In order to control for the option that the rats used auditory cues stemming from the motion of the positioning tables that positioned the poles (although this information was minimal if anything, see section Materials and Methods), we ran a control session, in which the positioning tables behaved exactly the same as in the experimental trials, but the poles were missing. Rats performed at chance level when poles were not available (Figure 4E). Interestingly, in this case rats adapted within a few trials a strategy of making an early decision, as indicated by the head trajectories (Figure 4F), while also making long sequences of same-side decision (Figure 4E, long sequence of right choices; left/right choices are indicated in blue/red; see also Figure 6 and related text).

### Temporal and Spatial Cues Were Used for Pole Localization

We investigated the use of temporal and spatial cues in the Narrow configuration, and a single whisker condition. We focused on the single whisker condition where performance was stable, more data was collected comparing to other conditions (see Table 1, Groups 1-2), the number of variables is smaller (only a single whisker on each side could contact a pole), and where it was possible to score whisker-pole contacts with no ambiguity (compared to single row or full pad conditions). First, we tested the hypothesis that temporal cues are essential. If animals make a decision based on the temporal order of pole contacts (first contact = reference pole), we expect that wrong contact order (when the rat contacts the anterior pole first) will lead to a wrong decision. We define DTp-a as the time difference between the first contact of the whiskers with the posterior pole, and the first contact of the whiskers with the anterior pole, such that a positive delay represents a case in which the posterior pole was contacted earlier. DTp-a is therefore well defined only in trials with bilateral contacts. In the single whisker condition, bilateral contacts occurred in 99.5 or 83.5% for groups 1 (Staircase training) and 2 (Block training), respectively. This difference might reflect the fact that, on average, offsets were smaller in the Staircase configuration, or a difference in the discrimination strategies. We found no difference in the mean performance level between trials with DTp-a > 0 and trials with DTp-a < 0 in rats trained in the Staircase-paradigm, for either small offset (<median offset over the session) or large offset (>median) trials. This suggests that for this group, a temporal cue was not crucial for discrimination.

In Block-paradigm trained rats, performance at large offsets (≥14 mm) did not depend on DTp-a sign, but for small offsets (≤10 mm), rats performed significantly better with DTp-a > 0 (*P* = 8⋅10^–5^, *t*-test) and at chance level with DTp-a < 0 (Figure 5A), suggesting that for small offsets the temporal order of whisker-pole contact was a crucial discriminative cue. Looking at the performance for negative and positive DTp-a as a function of offset for the same trials as in Figure 5A, we see that the smaller the offset is, the larger the effect of DTp-a sign is on performance (Figure 5B). For the smallest offset of 6mm, the performance when DTp-a < 0 is lower than chance level, suggesting that rats rely strongly on the temporal cue at this offset. We further observed that the fraction of trials with multiple whisker-reference pole contact was larger for small offsets (Figure 5C), suggesting an active sensing strategy, likely attempting to increase signal-to-noise ratio by averaging left-right differences over multiple bilateral contacts. Note that here rats have a single bilateral whisker, therefore multiple contacts mean more than one contact with a single whisker, rather than contacts with multiple whiskers. If the animal aims to compare the time-of-contact between poles, we expect protraction to cease only when the whisker at the anterior side contacts a pole, predicting larger whisking amplitude for large offsets, as indeed observed (Figure 5D). Rats performed significantly better when contacting the reference pole only, than when contacting the anterior pole only (Figure 5E, *P* < 0.01 for both 6 and 10 mm), consistent with the idea that rats assumed that the first contact is with the reference pole, and no contralateral contact is reflecting a large offset.

To test whether rats also used the absolute pole location for discrimination (rather than using relative spatial/temporal cues only), we trained three rats (out of the six rats in Group 2 – Narrow, Block) in a non-fixed reference configuration. Here, the offset was kept at 17 mm, the side of the reference pole (/reward) was chosen randomly as before, and the absolute location of the reference pole in the posterior-anterior axis was chosen randomly between three arrangements (‘unfixed reference’), as illustrated at the top of Figure 5F (arrow indicates the direction from entrance line to sipper line). All three rats performed better before changing from fixed to unfixed reference (*P* = 0.004, when comparing all trials in the last two fixed-reference sessions and all trials in the two first non-fixed reference session), indicating that the rats used the absolute pole location rather than only the relative position.

The lower performance in the ‘proximal’ poles’ arrangement (were the poles are closest to the entrance) for all 3 rats despite a similar offset of 17 mm in all three arrangements, could reflect a noisier measure of DTp-a in this arrangement (for 1 rat, blue in Figure 5F, the performance increase linearly with pole distance from entrance). We found that mean DTp-a was indeed lower in the ‘proximal’ arrangement (79 ms) comparing to mean DTp-a in the ‘original’ and ‘distal’ arrangements (101.4 and 111.4 ms; *P* = 0.007, two-sample *t*-test between ‘proximal’ and ‘original’-‘distal’ combined). As the offset was similar across the three arrangements, we hypothesized that the difference in DTp-a distribution between the conditions comes as a result of differences in head direction at the time of contact between the three arrangements. The bridge outside the entrance (see Figures 1A,B, rats walk on this bridge from entrance to sippers and back) gives a directional cue, that might be too short in the ‘proximal’ case. To directly test the effect of head trajectory on performance, we measured a ‘curving index’ (CI), defined as the ratio between the length of the head trajectory from entrance to sipper, and a straight line that connects the start/end-point in the trajectory (Figure 5G, see section Materials and Methods). Large CI indicates a non-straight pathway, which may result a noisy DTp-a. We found that, as expected, rats with smaller CI performed better (Figure 5H). Lastly, rats exhibited different head trajectories in hit versus miss trials (Figure 5I, single-whisker rats). Interestingly, the turning point was earlier on average in miss trials, suggesting some prior the rats had in the miss trials. This observation is consistent with the idea of an animal bias that exists at the beginning of each trial. We suggest that this bias depend on the stimuli and decisions in previous trials (see next section and Figure 6).

### Rat Decisions Were Affected by Choice and Success in Previous Trials

Motivated by previous studies (Carvell and Simons, 1990; Shuler et al., 2002; Raviv et al., 2012; Akrami et al., 2018; Campagner et al., 2019), we also tested if rat decision was affected by sensory history (Figure 6) in a way that depends on whether the rat does or does not rely on whiskers for localization. To test the effect of previous trials on rat decision, we looked at two groups of rats: group 2 (6 rats, Narrow arrangement, Block training) and Group 4 (3 rats, Wide arrangement, Staircase training, see Table 1), all rats in both groups having a single whisker (on each side). In the Wide configuration (Group 4) single whisker rats often did not contact the poles with the whiskers (see e.g., Supplementary Movie S4) and performance was typically poorer than that of Group 2 rats. Rat success, in this condition, was similar when whiskers contacted both poles (bilateral-contact) and when no whisker touched any pole (74% success rate in both cases), suggesting that in this condition (Wide, Single-whisker) rats relied strongly on body contacts. Success level was higher in trials that immediately followed a hit trial in Group 2 (Figure 6A, *P* < 0.001, bootstrap and Bonferroni correction for multiple-comparisons), with a smaller difference for large offsets. The probability for repeating the same choice (left/right) as in the previous trial was higher than chance for all offsets (Figure 6B, *P* < 0.001) and higher following a miss trial (significant for 10 and 14 mm, but not for 17 mm after multiple-comparison correction). In Group 4, performance was also higher following hit trials (Figure 6C, *P* < 0.001), and the probability for repeating the previous choice was different than chance (Figure 6D), with probability >0.5 to repeat the same choice following hit (*p* < 0.001 for all offsets), and probability <0.5 to repeat the same choice following miss (*p* = 0.035 for all offsets). This suggests that rats in this group were biased to go back to the side where reward was presented in the previous trial. To sum, rat decision was affected by previous trials. In the Narrow configuration (were whiskers were used to inform the decision, at least in low offsets), rats tended to repeat the same choice for multiple trials. In the wide configuration, were rats did not rely on whiskers for the decision and the performance was lower, rats were biased to go to the sipper that contained the reward in the previous trial (a Bayesian model is shown in the Supplementary Materials). The use of body (rather than whiskers) for discrimination therefore not only reduced the animal performance (Figures 2, 3), and delayed the decision (Figure 4), but also caused a bias toward going to the location of the reward in the previous trial.

## Discussion

This study addressed the generalization of object location. We show that rats that were trained to localize objects with their whiskers can transfer the task to other body parts – primarily trunk and ears – termed here “body.” This transfer was quick (within one session) in cases where body contacts occurred before whisker trimming, and slower (after one session) otherwise (compare Figure 2C and Figure 3C). Overall, although rats could solve the task with several body parts they relied on the whiskers, probably because, as we show, whisker-based localization occurred earlier (Figure 3) and at higher resolution (Figure 2). These findings are consistent with the rapid linking between whisker and head palpation of a given object, observed in any freely moving rodent behavior (e.g., Sherman et al., 2017). Such a rapid transfer can occur when the whisker and head coordinate frames are continuously linked.

Tactile object localization with non-whisker body parts has not been studied before as most of localization studies were done with head-fixed or nose-pocking rodents (Ahissar and Knutsen, 2008; Knutsen and Ahissar, 2009; O’Connor et al., 2010; Kleinfeld and Deschênes, 2011; Bush et al., 2016; Cheung et al., 2019). The use of freely moving rats here allowed the examination of their behavior in conditions that are closer to their natural behavior. As a result, we were able to show the preference of whisker-based localization and point on several reasons for this preference, primarily localization resolution and earlier processing. Part of the superiority of whisker-based localization can be accounted for by the fact that rats can rapidly repeat object contacts with whiskers, by simply executing another cycle of whisking (Figure 5C), a repetition that cannot be easily done with other body parts. While visual scoring of pole-whisker contact was not ambiguous in the case of a single row or a single whisker configuration, pole-ear contact was typically very brief, and it was often hard to determine whether such contact occurred (see e.g., Supplementary Movie S6). Rats never used the paws to contact a pole, as they were walking on an elevated bridge in the task area.

Sensory organs are embedded within motor-sensory-motor loops – anatomical loops through which the movements of a sensory organ induce sensory events that modify the movements of the same sensory organ (Ahissar et al., 2015; Ahissar and Assa, 2016). In general, the differences between whisker-based and head- or body-based localization can be attributed to the differences between the accuracy and resolution of the coding schemes employed by the specific motor-sensory-motor loops engaged in the task. As described in the Introduction, the whiskers can code object location using at least two coding schemes – the orthogonal (rate-time) and the morphological (motor-sensory). While the ears may employ a low-resolution version of both schemes, the trunk can probably employ only a low-resolution version of the orthogonal coding scheme. The lower resolution in these cases emerge not only because of sensory low-resolution, but, not less, from a lower resolution of motor control. These differences may account for the whiskers-body preference order observed here and further predict an ear-trunk preference. Nevertheless, our results suggest that despite resolution differences, the coding schemes that are relevant to different body parts may preserve consistent principles.

Animal decision was based not only on tactile cues available during the trial, but also on trial history (see Figure 6, Supplementary Figure S1, and Supplementary Materials). While relying on trial history was not an optimal strategy in our paradigm, its use by our rats suggest that it is a beneficial strategy in natural environments. Our data suggests that the effect of last trials on current decision was affected by the geometrical configuration (Narrow/Wide). This suggests that the tactile cues used by the rat (based on whiskers or body) not only affect the decision timing and accuracy, but also the weight of recent history on current decision.

We used two training procedures – Staircase and Block. The staircase procedure was used in order to find the localization threshold – the minimal detectable difference between the two poles. The block procedure was used to assess the psychometric performance – the mean success rate at each offset. Each rat was trained with one procedure only. Thus, the evaluation of the transfer of learning was always done by comparing performance in the same procedure. Rats that were trained in the Staircase paradigm achieved a threshold of 3.6 mm with a single whisker, and this threshold increased to 5.5 mm following the trimming of the last whisker (Figure 2B). When trained in the Block paradigm, trained rats did significantly better with a single whisker than with no whiskers in 6–10 mm offsets (Figure 3A). Thus, with both assessment methods, whisker-based localization yielded better spatial resolution.

With both training procedures, the freely moving condition allowed the rats to select their preferred sensory cues – here we focused on temporal and spatial cues (see Introduction). We show here that rats can rely on either spatial or temporal cues, depending on training, and that both can be generalized across body parts. In our paradigm, the choice of using temporal or spatial cues depended on the training procedure (Staircase vs. Block). Whereas with Staircase training the temporal cue was not key for rat decision, with Block training, temporal cues where essential at small offsets. Interestingly, for the Block-trained group, the number of whisker pole contacts (with the reference pole) was higher for small offsets, possibly indicating the use of multiple contacts for averaging out noise, when temporal cues are used (Figure 5).

Overall, high-speed tracking of freely moving rats during a localization task revealed the preference of rats to use their whiskers for localization, the superiority of whisker-based localization, and the surprising ability of rats to localize objects at somewhat lower resolution by body contacts.

## Author Contributions

DD designed and performed the experiments and analyzed the data. ES and EA mentored the experimental design, data analysis and modeling. DD and EA co-wrote the manusctipt.

## Conflict of Interest

The authors declare that the research was conducted in the absence of any commercial or financial relationships that could be construed as a potential conflict of interest.

## Footnotes

1 http://bwtt.sourceforge.net, vAIB, 2010

## References

[B1] Ahissar E. Assa E. (2016). Perception as a closed-loop convergence process. *eLife* 5:e12830. 10.7554/eLife.12830 27159238 PMC4913359

[B2] Ahissar E. Knutsen P. M. (2008). Object localization with whiskers. *Biol. Cybern.* 98 449–458. 10.1007/s00422-008-0214-4 18491159

[B3] Ahissar E. Knutsen P. M. (2011). Vibrissal location coding. *Scholarpedia* 6:6639 10.4249/scholarpedia.6639

[B4] Ahissar E. Shinde N. Haidarliu S. (2015). Systems neuroscience of touch. *Scholarpedia* 10:32785 10.4249/scholarpedia.32785

[B5] Akrami A. Kopec C. D. Diamond M. E. Brody C. D. (2018). Posterior parietal cortex represents sensory history and mediates its effects on behaviour. *Nature* 554 368–372. 10.1038/nature25510 29414944

[B6] Bagdasarian K. Szwed M. Knutsen P. M. Deutsch D. Derdikman D. Pietr M. (2013). Pre-neuronal morphological processing of object location by individual whiskers. *Nat. Neurosci.* 16 622–631. 10.1038/nn.3378 23563582

[B7] Bale M. R. Campagner D. Erskine A. Petersen R. S. (2015). Microsecond-scale timing precision in rodent trigeminal primary afferents. *J. Neurosci.* 35 5935–5940. 10.1523/JNEUROSCI.3876-14.2015 25878266 PMC4397594

[B8] Birdwell J. A. Solomon J. H. Thajchayapong M. Taylor M. A. Cheely M. R. Towal B. (2007). Biomechanical models for radial distance detection by rat vibrissae. *J. Neurophysiol.* 98 2439–2455. 10.1152/jn.00707.2006 17553946

[B9] Boubenec Y. Shulz D. E. Debregeas G. (2012). Whisker encoding of mechanical events during active tactile exploration. *Front. Behav. Neurosci.* 6:74. 10.3389/fnbeh.2012.00074 23133410 PMC3490139

[B10] Bush N. E. Solla S. A. Hartmann M. J. (2016). Whisking mechanics and active sensing. *Curr. Opin. Neurobiol.* 40 178–188. 10.1016/j.conb.2016.08.001 27632212 PMC5312677

[B11] Campagner D. Evans M. H. Bale M. R. Erskine A. Petersen R. S. (2016). Prediction of primary somatosensory neuron activity during active tactile exploration. *eLife* 5:e10696. 10.7554/eLife.10696 26880559 PMC4764568

[B12] Campagner D. Evans M. H. Chlebikova K. Colins-Rodriguez A. M. Loft S. Fox S. (2019). Prediction of choice from competing mechanosensory and choice-memory cues during active tactile decision making. *J. Neurosci.* 39 2217–2218. 10.1523/JNEUROSCI.2217-18.2019 30850514 PMC6520515

[B13] Campagner D. Evans M. H. Loft M. S. Petersen R. S. (2018). What the whiskers tell the brain. *Neuroscience* 368 95–108. 10.1016/j.neuroscience.2017.08.005 28843998

[B14] Carvell G. E. Simons D. J. (1990). Biometric analyses of vibrissal tactile discrimination in the rat. *J. Neurosci.* 10 2638–2648. 10.1523/jneurosci.10-08-02638.1990 2388081 PMC6570272

[B15] Cheung J. Maire P. Kim J. Sy J. Hires S. A. (2019). The sensorimotor basis of whisker-guided anteroposterior object localization in head-fixed mice. *Curr. Biol.* 29 3029–3040. 10.1016/j.cub.2019.07.068 31474537 PMC6771421

[B16] Curtis J. C. Kleinfeld D. (2009). Phase-to-rate transformations encode touch in cortical neurons of a scanning sensorimotor system. *Nat. Neurosci.* 12 492–501. 10.1038/nn.2283 19270688 PMC2863011

[B17] Deutsch D. Pietr M. Knutsen P. M. Ahissar E. Schneidman E. (2012). Fast feedback in active sensing: touch-induced changes to whisker-object interaction. *PLoS One* 7:e44272. 10.1371/journal.pone.0044272 23028512 PMC3445569

[B18] Diamond M. E. von Heimendahl M. Knutsen P. M. Kleinfeld D. Ahissar E. (2008). ‘Where’ and ‘what’ in the whisker sensorimotor system. *Nat. Rev. Neurosci.* 9 601–612. 10.1038/nrn2411 18641667

[B19] Kleinfeld D. Ahissar E. Diamond M. E. (2006). Active sensation: insights from the rodent vibrissa sensorimotor system. *Curr. Opin. Neurobiol.* 16 435–444. 10.1016/j.conb.2006.06.009 16837190

[B20] Kleinfeld D. Deschênes M. (2011). Neuronal basis for object location in the vibrissa scanning sensorimotor system. *Neuron* 72 455–468. 10.1016/j.neuron.2011.10.009 22078505 PMC3971931

[B21] Knutsen P. M. Ahissar E. (2009). Orthogonal coding of object location. *Trends Neurosci.* 32 101–109. 10.1016/j.tins.2008.10.002 19070909

[B22] Knutsen P. M. Derdikman D. Ahissar E. (2005). Tracking whisker and head movements in unrestrained behaving rodents. *J. Neurophysiol.* 93 2294–2301. 10.1152/jn.00718.2004 15563552

[B23] Knutsen P. M. Pietr M. Ahissar E. (2006). Haptic object localization in the vibrissal system: behavior and performance. *J. Neurosci.* 26 8451–8464. 10.1523/jneurosci.1516-06.2006 16914670 PMC6674338

[B24] Moore J. D. Lindsay N. M. Deschênes M. Kleinfeld D. (2015). Vibrissa self-motion and touch are reliably encoded along the same somatosensory pathway from brainstem through thalamus. *PLoS Biol.* 13:e1002253. 10.1371/journal.pbio.1002253 26393890 PMC4579082

[B25] O’Connor D. H. Clack N. G. Huber D. Komiyama T. Myers E. W. Svoboda K. (2010). Vibrissa-based object localization in head-fixed mice. *J. Neurosci.* 30 1947–1967. 10.1523/JNEUROSCI.3762-09.2010 20130203 PMC6634009

[B26] Pammer L. O’Connor D. H. Hires S. A. Clack N. G. Huber D. Myers E. W. (2013). The mechanical variables underlying object localization along the axis of the whisker. *J. Neurosci.* 33 6726–6741. 10.1523/JNEUROSCI.4316-12.2013 23595731 PMC3733083

[B27] Quist B. W. Seghete V. Huet L. A. Murphey T. D. Hartmann M. J. (2014). Modeling forces and moments at the base of a rat vibrissa during noncontact whisking and whisking against an object. *J. Neurosci.* 34 9828–9844. 10.1523/JNEUROSCI.1707-12.2014 25057187 PMC4107402

[B28] Raviv O. Ahissar M. Loewenstein Y. (2012). How recent history affects perception: the normative approach and its heuristic approximation. *PLoS Comput. Biol.* 8:e1002731. 10.1371/journal.pcbi.1002731 23133343 PMC3486920

[B29] Saraf-Sinik I. Assa E. Ahissar E. (2015). Motion makes sense: an adaptive motor-sensory strategy underlies the perception of object location in rats. *J. Neurosci.* 35 8777–8789. 10.1523/JNEUROSCI.4149-14.2015 26063912 PMC6605211

[B30] Severson K. S. Xu D. Van de Loo M. Bai L. Ginty D. D. O’Connor D. H. (2017). Active touch and self-motion encoding by merkel cell-associated afferents. *Neuron* 94 666.e9–676.e9. 10.1016/j.neuron.2017.03.045 28434802 PMC5528144

[B31] Sherman D. Oram T. Harel D. Ahissar E. (2017). attention robustly gates a closed-loop touch reflex. *Curr. Biol.* 27 1836–1843. 10.1016/j.cub.2017.05.058 28602655

[B32] Shuler M. G. Krupa D. J. Nicolelis M. A. (2002). Integration of bilateral whisker stimuli in rats: role of the whisker barrel cortices. *Cereb. Cortex* 12 86–97. 10.1093/cercor/12.1.86 11734535

[B33] Solomon J. H. Hartmann M. J. (2006). Biomechanics: robotic whiskers used to sense features. *Nature* 443:525. 10.1038/443525a 17024083

[B34] Szwed M. Bagdasarian K. Ahissar E. (2003). Encoding of vibrissal active touch. *Neuron* 40 621–630. 10.1016/s0896-6273(03)00671-8 14642284

[B35] Szwed M. Bagdasarian K. Blumenfeld B. Barak O. Derdikman D. Ahissar E. (2006). Responses of trigeminal ganglion neurons to the radial distance of contact during active vibrissal touch. *J. Neurophysiol.* 95 791–802. 10.1152/jn.00571.2005 16207785

[B36] Towal R. B. Quist B. W. Gopal V. J. Solomon H. Hartmann M. J. (2011). The morphology of the rat vibrissal array: a model for quantifying spatiotemporal patterns of whisker-object contact. *PLoS Comput. Biol.* 7:e1001120. 10.1371/journal.pcbi.1001120 21490724 PMC3072363

[B37] Uexkull J. V. (1926). *Theoretical Biology.* London: K Paul, Trench, Trubner & co. ltd.

[B38] Urbain N. Salin P. A. Libourel P.-A. Comte J.-C. Gentet L. J. Petersen C. C. (2015). Whisking-related changes in neuronal firing and membrane potential dynamics in the somatosensory thalamus of awake mice. *Cell Rep.* 13 647–656. 10.1016/j.celrep.2015.09.029 26489463

[B39] Voigts J. Herman D. H. Celikel T. (2015). Tactile object localization by anticipatory whisker motion. *J. Neurophysiol.* 113 620–632. 10.1152/jn.00241.2014 25339711

