# Supplementary figures and images for: Generalization of Object Localization From Whiskers to Other Body Parts in Freely Moving Rats
Source: Front Integr Neurosci. 2019 Oct 31;13:64. doi: 10.3389/fnint.2019.00064 (PMC6839537; doi:10.3389/fnint.2019.00064)

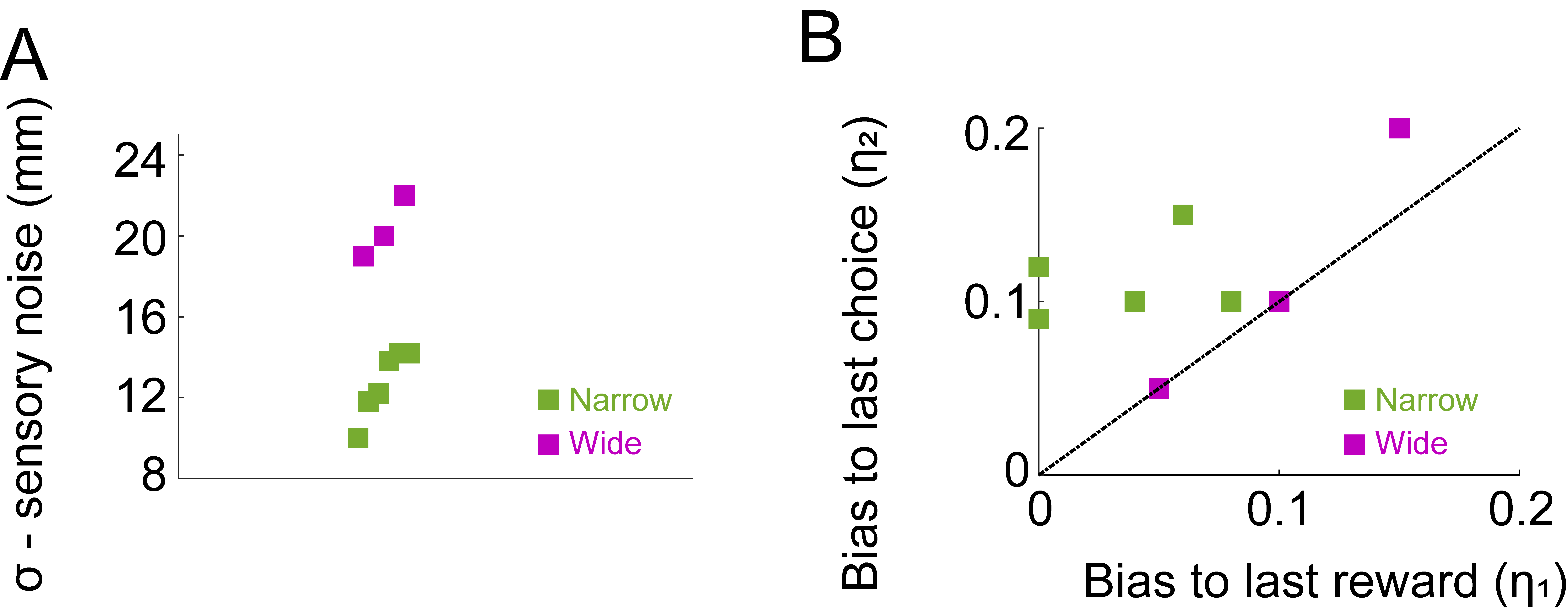

Supplement: Supplementary file 1 [file Image_1.TIF]
