# Supplementary material for: Generalization of Object Localization From Whiskers to Other Body Parts in Freely Moving Rats
Source: Front Integr Neurosci. 2019 Oct 31;13:64. doi: 10.3389/fnint.2019.00064 (PMC6839537; doi:10.3389/fnint.2019.00064)
Supplement: Supplementary file 2 [file Table_1.pdf]

SUPPLEMENTARY MATERIAL

Supplementary methods - Bayesian model for sensory history

Animal decision in a given trial was modeled as sampling from a Gaussian distribution (s - sampling value) with mean around the actual offset s' (equations 1-3 below), and comparing the sample with a threshold (θ) that change between trials, based on previous trials (equation 4).

We used two models for the threshold. In model 1, the threshold is updated based on the side of the last reward. If, for example, the reward in the current trial is on the right side (whether the animal made a left or a right choice), θ will change in the direction that will make a higher probability for right choice in the next trial. In model 2, the threshold is updated based on animal choice (left or right), regardless of the reward side. There are two free parameters in each model, σ – the width of the Gaussian, representing sensory noise, and η, the rate of threshold update (η_1 or η_2 for models 1 or 2). The likelihood of a specific sequence of choices made by the rat given a specific set of model parameters was calculated using the loglikelihood of the animal choices (equation 5). For each rat (six rats from Group 2 – Narrow, Block; three rats from Group 4 – Wide, Staircase) and model, the set (σ, η) that gives the highest likelihood value was found (see Figure S1A-B). Below is the detailed derivation.

Given that the actual distance between the poles in a given trial was s, the animal perceived s' is a noisy representation around s with variance σ^2

(1) $$p(s'|s) = N(s - s'; \sigma^2)$$

σ represents the animal sensory noise.

The animal had to decide if *s* is positive or negative, and we assume that it did so by comparing the perceived distance with some threshold θ. The probability that the animal would think the distance is positive equals

(2) $$L^+(s;\theta) = p(s' > \theta|s) = \int_\theta^\infty N(s - s'; \sigma^2)\, ds' = \int_\theta^\infty \frac{1}{\sigma\sqrt{2\pi}} e^{-\frac{(s-s')^2}{2\sigma^2}} ds' = 1 - \int_{-\infty}^\theta \frac{1}{\sigma\sqrt{2\pi}} e^{-\frac{(s-s')^2}{2\sigma^2}} ds' = \frac{1}{2}\left(1 - \mathrm{erf}\left(\frac{\theta - s}{\sigma\sqrt{2}}\right)\right)$$

and negative

(3) $$L^-(s;\theta) = p(s' \le \theta|s) = 1 - L^+(s;\theta)$$

we assume that the threshold θ_t changed by a constant η after each trial so that -

(4) $$\theta_t = \theta_{t-1} + \eta g_{t-1}$$

Where g_{t-1} is the last reward side in model 1 and the last choice side in model 2.

For each model, we found the pair of parameters (σ, η) that best fit to the choices of each individual, by finding the maximum of the log likelihood of the animal choices $c(t)$ over trials:

(5) $$LL = \sum_t \log L^{c(t)}(s(t); \theta_t)$$

Supplementary results - A Bayesian model predicts animal choice and success based on previous trials

Rat decision in a given trial was effected by the reward position and animal choice in previous trials (see Figure 6).

We used two Bayesian models to predict rat decision based on sensory cues and previous trials. In model 1, the rat decision in a given trial was biased towards the last reward side. In model 2, the bias was to the last animal choice (see Methods for more details).

For each rat, we fitted both models separately, using maximum likelihood between the predicted and actual rat choices (in all sessions with a single whisker; see Methods). Figure S1A shows the value of σ (best fit) found for each rat (Group 2 in green, Group 4 in purple) from model 2 (both models gave the same sensory noise for a given rat ±1 mm). Smaller sensory noise was found in Group 2 animals comparing to Group 4 animals (Figure S1A). This might reflect the fact that whiskers were used for localization in Group 2 (Figure 5), while the success in Group 4 rats did not depend on whisker-pole contacts. Figure S1B shows the best fit for η_1 and η_2 (from models 1 and 2) for each rat. For all rats in Group 2 (Figure S1B, green), the bias to the last choice was stronger than the bias to the last reward, consistent with long-bouts of choice-repeat. In Group 4 rats had both types of biases with around the same weights (Figure 6F, purple). Interestingly, when the sensory noise was larger (Wide vs Narrow), rats relied more on the reward side in previous trials (Figure S1B).

Supplementary figures

Figure S1 - Dependence of decisions on previous trials: Bayesian model

A. Sensory noise (parameter 1). **B**. Bias for last choice (parameter 2) versus the bias to last reward (parameter 3) for all rats in the Narrow-Block Group. All rats in A-B had a single whisker (bilateral) only. Figure S1 is related to figure 6.

Supplementary movies

Movie S1 (FieldCamera_SingleTrial.wmv) – An example trial from a field camera. Rat drinks mango juice in the arena, after returning from a previous trial. The juice in the arena is given at the end of each trial regardless of animal success, and always at the same port, encouraging the rat to return fast to the arena at the end of each trial, whether a hit or a miss trial. The rat waits for the bridge to open, goes out to the task area, walks on an elevated bridge and turns to one of two sippers (in this case to the left sipper), gets a reward (mango juice) if a correct choice was made and no reward if not. Then the rat comes back into the arena to get juice, and the door is closing. The whole procedure is fully automated. A sensor detects when the rat is going through the door, and the door stays open until the rat gets back into the arena. The motors move to a reset position and then back to the next position when the rat is in the arena (so motor-noise exists whether the pole-configuration did or did not change between trials). The back-light seen in the task area is an IR back-light (940 nm, a wave-length that is not perceived by our rats; see methods)) used for tracking. IR sensitive field camera was used to take this video; additional, high-speed, camera was used for tracking. The movie runs at real time.

Movie S2 (TopCamera_NarrowStaircase_SingleWhisker.mp4) – Top camera, filming at 500 Hz. A rat from Group 1 (Narrow poles-sipper configuration, Staircase paradigm, single whisker; same as in figure 1A). Rat contacted both poles with its whiskers, before making a choice. Here the rat made a correct choice [left sipper – same side as the reference (posterior) pole], and reward was given upon licking the sipper. The Narrow configuration was designed such that rats contact the poles with the body on the way to the sippers.

Movie S3 (TopCamera_WideStaircase_FullPad.mp4) – A rat from Group 3 (Wide poles-sipper configuration, Staircase paradigm, full pad; same as in figure 1B). The rat made a correct left choice. Note that the rat did not contact any pole with its body before approaching the reward sipper. The Wide configuration was designed to allow drinking from a sipper without any body-pole contact.

Movie S4 (TopCamera_BodyContact.mp4) – A rat from Group 4 (Wide poles-sipper configuration, Staircase paradigm, single bilateral whisker (C2)). With a single whisker, at the Wide configuration, rats often (as in this movie) did not contact any pole with the whiskers before getting to the sippers. Here the rat made a correct right choice after contacting a single pole with the body.

Movie S5 (TopCamera_MultipleContacts.mp4) – A rat from Group 1 (Narrow poles-sipper configuration, Staircase paradigm, single row (row C)). Here the rat made three whisking cycles, the first before contact and two with bilateral contacts, before making a (left, correct) choice.

Movie S6 (TopCamera_Trunk and ear, example 1.mp4) – A rat from Group 2 (Narrow pole-sipper configuration, Block paradigm, single row (row C)). In this example, the rat contacted the right pole with the right ear. The rat contacts the left pole with hair on the left trunk, and it is hard to judge if the right ear contacted the pole.

Movie S7 (TopCamera_Trunk and ear, example 2.mp4) – A rat from Group 2 (Narrow pole-sipper configuration, Block paradigm, single row (row C)). In this example, the rat first contacted both poles with the whiskers, following light contact between the right pole and the right ear and trunk and a light left trunk-pole contact. Trunk and ear contacts with the poles vary between trials in sequence (left/right, trunk/ear) and in contact strength (light or firm contact).

Movie S8 (TopCamera_No Whiskers.mp4) – A rat from Group 2 (Narrow pole-sipper configuration, Block paradigm, no whiskers). In this example, the rat is contacting the reference/posterior pole and the anterior pole with the ears and trunk, before making a correct decision.
